# Highly Porous and Superabsorbent Biomaterial Made of Marine-Derived Polysaccharides and Ascorbic Acid as an Optimal Dressing for Exuding Wound Management

**DOI:** 10.3390/ma14051211

**Published:** 2021-03-04

**Authors:** Vladyslav Vivcharenko, Michal Wojcik, Krzysztof Palka, Agata Przekora

**Affiliations:** 1 Department of Biochemistry and Biotechnology, Medical University of Lublin, Chodzki 1 Street, 20-093 Lublin, Poland; vlad.vivcharenko@gmail.com (V.V.); michal.wojcik@umlub.pl (M.W.); 2 Department of Materials Science and Engineering, Faculty of Mechanical Engineering, Lublin University of Technology, Nadbystrzycka 36, 20-618 Lublin, Poland; k.palka@pollub.pl

**Keywords:** biocompatibility, fibroblasts, biodegradation, mechanical test, chitosan, agarose, wound dressing

## Abstract

There are many modern wound dressings that have promising properties for repairing skin damage. However, due to various types of wounds and the problems they cause, there is still a great demand for new, effective healing strategies. The aim of this study was to create superabsorbent wound dressing made of marine-derived polysaccharides (agarose and chitosan) using the freeze-drying method. The secondary goal was its comprehensive evaluation for potential use as an external superabsorbent bandage for wounds with high exudation. Due to the well-known positive effect of ascorbic acid (vitamin C) on the healing process, biomaterial enriched with vitamin C was prepared and compared to the variant without the addition of ascorbic acid. It was shown that the produced foam-like wound dressing had a very porous structure, which was characterized by hydrophilicity, allowing a large amount of human fluids to be absorbed. According to in vitro tests on human fibroblasts, biomaterial was nontoxic and supportive to cell proliferation. Vitamin C-enriched dressing also had the ability to significantly reduce matrix metalloproteinase-2 production and to promote platelet-derived growth factor-BB synthesis by fibroblasts, which is desired during chronic wound treatment. The material has features of the eco-friendly wound care product since it was made of naturally-derived polysaccharides and was proved to be biodegradable. Importantly, despite degradable character, it was stable in the chronic and infected wound microenvironment, maintaining high integrity after 8-week incubation in the enzymatic solutions containing lysozyme and collagenases. The obtained results clearly showed that developed biomaterial possesses all necessary features of the external dressing for the management of exudate from both acute and chronic non-healing wounds.

## 1. Introduction

Unique properties of the biomaterials, such as biocompatibility, controlled degradation and porous network with good mechanical features, have enabled their broad application in tissue engineering and regenerative medicine [1]. In recent years, scientists have mainly focused on the production of composite and hybrid biomaterials by combining different polymers. In contrast to the single constituent materials, the second ones possess improved mechanical properties and show better cellular response increasing their biocompatibility [2]. The modern concept of wound repair includes its accelerated healing by application of bioactive dressings made of synthetic or natural polymers and/or growth factors. The main features of the dressings used in the process of skin regeneration include: stimulation of the epithelialization, moisture retention, inhibition of microbial infections, and reduction of exudate at the wound bed [3]. An additional advantage of the biomaterial used as an external wound dressing is its ability to deliver drugs to the wound bed, increasing the effectiveness of the treatment and accelerating the healing process. In the case of infected chronic wounds, the long term antimicrobial effect may be obtained by the delivery of various active substances and nanoparticles to the wound bed [4]. Nevertheless, dressing materials with drugs incorporated should meet several important criteria, which include gradual drug release from the structure of the biomaterial or no reduction in drug effectiveness due to its immobilization [5]. Scientists are still searching for optimal wound dressing based on natural or synthetic compounds that would have all of the mentioned features, providing optimal conditions for skin tissue repair [6]. The category of natural biodegradable polymers that are used in the dressing production includes: a variety of proteins (e.g., collagen, fibrin, fibroin), marine-derived polysaccharides (e.g., chitosan, agarose, alginate), and other naturally occurring polymers (e.g., cellulose, starch, hyaluronic acid etc.). Among the biodegradable synthetic materials used in biomaterials engineering are poly-glycolic acid (PGA), poly lactic acid (PLA), polycaprolactone (PCL) and poly lactic-co-glicolide (PLGA) copolymers [7].

Chitosan is a well-known polysaccharide with relevant features that enable its potential use in biomedical application [8]. The most important features include its antimicrobial properties, biocompatibility, non-toxicity, and also biodegradability [9,10]. Due to its antimicrobial properties, wound dressing made of chitosan can be produced both alone and with the addition of antimicrobial nanoparticles, antibiotics or natural extracts that increase its effectiveness in heavily infected wounds [9]. The main restrictions of the chitosan use are related to the loss of its mechanical properties by water absorption and subsequent swelling. Consequently, it was necessary to use chemical crosslinkers to stabilize chitosan structure [11]. The addition of an appropriate concentration of agarose not only improves the mechanical properties of chitosan-based biomaterials, but also allows for the imitation of soft tissue mechanics. Hydrogen bonds resulting from the preparation of agarose/chitosan hydrogel exclude the need for use of toxic crosslinking agents [8].

External dressings for skin wounds should remove excessive exudate, provide oxygen and adequate humidity necessary for primary wound healing [12]. All mentioned features highly depend on the microstructure of the dressing material. Extremely porous and absorbent biomaterials are ideal for wounds with high levels of exudate [13]. Among all known techniques for the production of porous biomaterials in engineering of biomaterials, the most frequently used methods are freeze-drying, porogen-leaching, and gas-foaming. The profitability and simplicity of the above-mentioned methods allow us to obtain a highly macroporous structure of the material [14].

It is assumed that the nutritional status of micro- and macronutrients is essential for both appearance and skin health. To prove the above statement, it is enough to consider the diseases associated with vitamin deficiency [15]. Vitamin C possesses antioxidant features that prevent scar formation and skin damages, improving skin barrier functionality and moisture content. It is the best described and most considerable compound supporting wound healing by promotion of cell proliferation and collagen synthesis [16].

The aim of this work was to produce freeze-dried biomaterial composed of chitosan and agarose with incorporated vitamin C for potential application as a superabsorbent external wound bandage to manage exudate from both acute and chronic non-healing wounds. In the available scientific literature, there are already a few well-described biomaterials made of chitosan and agarose that were fabricated using freeze-drying production method to obtain biomaterials for soft tissue regeneration [2,17]. Nevertheless, the study presented here shows the new method that allows for the production of chitosan/agarose biomaterial in one effortless step by obtaining the optimal pH (slightly below 6) for skin cell growth and regeneration process. The fabrication method described here skips the necessity of sample neutralization and the rinse stage after the production process since the concentrations of polysaccharides and their solvents (acetic acid and sodium hydroxide) were chosen to prevent the precipitation and aggregation of chitosan—which precipitates at pH ≥ 6.5—and to obtain a desired slightly acidic pH of the resultant solution. Importantly, the production method of the porous chitosan/agarose wound dressing has been claimed in the Polish Patent Office (patent application no. P.430457).

Within this study, superabsorbent chitosan/agarose biomaterial was produced and submitted to a broad evaluation of its biomedical usefulness as external wound bandage via determination of exudate absorption capacity, wettability, porosity, mechanical properties, biodegradability, and biocompatibility in vitro.

## 2. Materials and Methods

### 2.1. Preparation of the Biomaterial

The 4% (*w/v*) agarose solution was prepared by dissolving agarose (low EEO, Sigma-Aldrich Chemicals, Warsaw, Poland) in 0.1% (*w/v*) sodium hydroxide (NaOH) (Avantor Performance Materials, Gliwice, Poland). The mixture was heated at 95 °C for 10 min. The 3% (*w/v*) chitosan solution was obtained by dissolution of chitosan (1.174 × 10^6^ Da, 73% deacetylation degree, National Marine Fisheries Research Institute, Gdynia, Poland) in 0.5% (*v/v*) acetic acid (CH_3_COOH) (Avantor Performance Materials, Gliwice, Poland). In order to completely dissolve the chitosan, the beaker with the solution was incubated for 24 h at 37 °C. After the incubation, both mixtures were mixed at the ratio 1:1. When the temperature of the obtained mixture lowered to the range of 40–45 °C, 3-O-ethyl-L-ascorbic acid solution (Sigma-Aldrich Chemicals, Warsaw, Poland) prepared in a phosphate buffered saline (PBS, Sigma-Aldrich Chemicals, Warsaw, Poland) was added. In the next step, the pH of the chitosan/agarose mass without and with the addition of vitamin C was measured and the mixture was spread on the polystyrene surface forming a layer with a thickness of 4 mm. Finally, prepared samples were frozen at −80 °C for 4 h and lyophilized in a vacuum of 6 × 10^2^ mbar (LYO GT2-Basic, SRK Systemtechnik GmbH, Riedstadt, Germany) for 18 h. After lyophilization, biomaterials without vitamin C and with the final concentrations of 100 µg/mL (CHN/A + 100) and 200 µg/mL (CHN/A + 200) of vitamin C were cut, weighed, and used for the tests. Table 1 summarizes sample designation and compositions. After the production process, the developed biomaterial (dry and hydrated) was visualized using a stereoscopic microscope (Olympus SZ61TR, Olympus Polska Sp. z o. o., Warsaw, Poland).

### 2.2. Basic Characterization: Wettability, Microstructural, and Mechanical Properties

To establish the wettability of the produced biomaterials, DSA 30 goniometer (Kruss GmbH, Hamburg, Germany) was used. Wettability was evaluated in the static contact angle method (sessile drop technique) using ultrapure water obtained from Milli-Q^®^ Water Purification System (Merck, Warsaw, Poland). The wetting behavior of the samples was calculated based on the measurements performed for four independent samples. A microcomputed tomography (microCT) (Skyscan 1174, Bruker MicroCT, Kontich, Belgium) was used in the evaluation of biomaterial porosity. The scan was performed with a resolution of 6.4 μm, obtaining the set of images taken every 0.5 degrees in the semi-full angle. Acquired set of 360 images was then reconstructed using NRecon (Bruker microCT, Kontich, Belgium) to obtain cross-sections useful for analysis. Binarization was performed using the Otsu algorithm. The total porosity was assessed on 2D sections using CTAnalyser software (Bruker microCT, Kontich, Belgium). In contrast, the pore size was assessed using the method proposed by Hildebrand and Ruegsegger [18], which uses the diameter of the largest sphere inscribed in the analyzed area; in this case, free space (porosity).

Autograph AG-X plus universal testing machine (Shimadzu, Kyoto, Japan) was used in the determination of the mechanical properties of the produced material in a tensile mode. Before the experiment, all samples (6 mm in width and 70 mm in length) were presoaked for 60 min in PBS. A total of four individual biomaterials were subjected to the mechanical test. The crosshead rate was 50 mm/min. Ultimate tensile strength (UTS) was calculated as the ratio of the highest recorded force value to the initial cross-section of the test sample. The ultimate elongation at break was determined as the sum of elastic and plastic deformations at the moment of fracture. The elastic modulus (Young’s modulus) was evaluated using Aramis Optical Strain system (Trilion Quality Systems, King of Prussia, PA, USA). The value of Young′s modulus was determined as the ratio of the stress increase in the strain range from 0.05% to 0.25%.

### 2.3. Biodegradation Test

A biodegradation test was performed to evaluate the stability of the biomaterial in different enzymes occurring in the chronic and infected wounds. The biomaterial was cut into small pieces weighting 20 ± 2 mg. Enzymatic degradation test was conducted using 3 different solutions: (1) 150 mg/L of type I collagenase (MMP-1) and 150 mg/L of type II collagenase (MMP-8) (Thermo Fisher Scientific, Waltham, MA, USA) dissolved in PBS (pH 7.4), (2) 1 g/L of lysozyme (Sigma-Aldrich Chemicals, Warsaw, Poland) dissolved in a phosphate buffer (pH 6.0), (3) PBS control solution (pH 7.4)—stability assessment and non-enzymatic degradation evaluation. Samples were placed in 15 mL falcons and filled to 10 mL with prepared solutions. Then, falcons were incubated in a thermal shaker for 56 days (60 rpm, 37 °C). The degree of degradation was assessed on the basis of changes in the pH and concentration of reducing sugars in the solutions. Therefore, 3 mL of each degradation solution was collected every 14 days and initial volumes of 10 mL were refilled with freshly prepared enzymatic or PBS solution. Then, pH and reducing sugars concentrations were measured in the collected samples. For this purpose, colorimetric 3,5-dinitrosalicylic acid-based method was used [19]. To include the dilution error, the following formula was used to calculate the concentration of reducing sugars in the collected samples:

(1) $$F_{n}=10c_{n}\;+\;\overset{n-1}{\underset{i=1}{\sum }}3c_{i}$$

where *F* is the final concentration of reducing sugars released into the degradation solution (µg), C is the concentration of the reducing sugars in the collected sample (µg/mL), and n corresponds to the number of measurement performed for the determination of reducing sugars concentration, i.e., *n* = 1—measurement 1 for sample collected after two weeks, *n* = 2—measurement 2 for sample collected after four weeks, etc.

After the test, samples were rinsed with deionized water to wash out the remains of degradation solutions, and lyophilized. After the lyophilization process, the top surface and cross-section of the samples were visualized using scanning electron microscope (SEM JEOL JCM-6000Plus, Tokyo, Japan). For this purpose, samples with a sprayed thin layer of gold (8 nm) were prepared using a high vacuum sputter. SEM images were obtained at an accelerating voltage of 5 kV in a high vacuum environment.

### 2.4. Exudate Absorption Capacity

Human blood serum and human blood plasma (obtained from Blood Bank, Lublin, Poland) were used to estimate the absorption capacity of the produced material. The materials were cut into small samples (4 mm × 4 mm) with an average weight mass of about 7 ± 1 mg. Samples were immersed in the physiological fluids for 24 h. The samples were weighed at specified time intervals (1 s, 2 s, 4 s, 8 s, 16 s, 30 s, 1 min, 2 min, 5 min, 10 min, 30 min, 1 h, 2 h, 24 h) and immersed back into fluids. Paper tissues were used to remove the excess of the fluids before weighting. The volume (mL) of the absorbed serum/plasma by 1 g of biomaterial was determined based on the Equation (2) [12]:

(2) $$V=\frac{m_{w}-m_{i}}{g\times m_{i}}$$

where *V* represents a volume of absorbed serum/plasma (mL), *m_w_* represents the wet sample weight (g) after immersion in blood serum or plasma, *m_i_* represents an initial weight of dry sample (g), and *g* is a plasma or serum density (g/mL). The maximum absorption capacity (*W_max_%*) expressed as a percentage of a weight increase after 24 h was also calculated according to the following Equation (3) [12]:

(3) $$W_{i}\%=\frac{W_{w}-W_{d}}{W_{d}}\times 100$$

where *W_w_* and *W_d_* are weights of the wet (after 24 h) and dry samples, respectively.

### 2.5. Vitamin C Release

To estimate the amount of vitamin C released from the structure of the biomaterials, a flow-through test in a closed-loop system was used. For this purpose, CHN/A +100 and CHN/A + 200 samples weighting 250 ± 2 mg were applied. The experiment was conducted in a drug release apparatus (USP4 Sotax, Donau Lab, Budapest, Hungary) with the elution medium (PBS) in the final volume of 50 mL. The circulated rate was 3 mL per min at 37 °C. Vitamin C concentration was evaluated in 0.5 mL samples collected at defined time intervals. The amount of the elution medium was refilled to the original volume with freshly prepared PBS after sample collection. UV-spectrophotometer with 252 nm wavelength (Genesys 6 UV-Vis, Thermo Fisher Scientific, Waltham, MA, USA) was used to determine the cumulative concentration of vitamin C. The absorbance values were measured and concentration was calculated using the calibration curve prepared for 3-O-ethyl-L-ascorbic acid solution (3.75–60 µg/mL).

### 2.6. Cell Culture Tests

Normal human skin fibroblasts (BJ cell line) obtained from American Type Culture Collection (ATCC, Teddington, UK) were used in the presented work. Human skin fibroblasts were cultured in a dedicated Eagle’s Minimum Essential Medium (EMEM, ATCC-LGC Standards, Teddington, UK). The culture medium also contained: 10% fetal bovine serum (FBS, Pan-Biotech GmbH, Aidenbach, Bavaria, Germany), streptomycin (100 µg/mL), and penicillin (100 U/mL), which were obtained from Sigma-Aldrich Chemicals (Warsaw, Poland). The cell line was cultured in the recommended by ATCC conditions: 37 °C, 5% CO_2_, and 95% air humidity.

#### 2.6.1. Cytotoxicity Test

Cytotoxicity of produced biomaterials was evaluated via determination of BJ fibroblasts viability after exposure to 24 h biomaterials extracts (test according to ISO 10993-5:2009) [20]. Extracts were prepared by incubation of the samples in the BJ culture medium (EMEM) for 24 h at 37 °C. Compared to the mentioned ISO standard, the biomaterial/medium ratio was reduced to 15 mg of the biomaterial per 1 mL of EMEM medium due to the high swelling tendency and absorption capacity of the porous materials. Human skin fibroblasts at a concentration of 2 × 10^4^ cells per well were seeded into 96-multiwell plates and cultured for 24 h. Then, the medium was replaced with the extracts obtained from biomaterials and polypropylene (negative control of cytotoxicity). The cells were incubated for a further 24 and 48 h. After incubation, MTT viability test was performed (Sigma-Aldrich Chemical, Warsaw, Poland) according to the procedure described previously [21]. The results were presented as the percentage of cell viability compared to the negative control (cells cultured with polypropylene extract; reference material in accordance with ISO 10993-12 [22]).

To evaluate human fibroblast viability in direct contact with the biomaterial, the cells were seeded directly on the CHN/A sample. A direct contact cytotoxicity test allowed also to determine cell attachment to the surface of the biomaterial. Small biomaterial samples (6 mm × 6 mm) were placed in a 48-multiwell plate and presoaked in fibroblasts culture medium. Then, 500 µL of BJ cells at a concentration of 2 × 10^5^ cells per mL were seeded directly on the biomaterial and cultured for 48 h at 37 °C. Live/Dead Double Fluorescent Staining Kit (Sigma-Aldrich Chemicals, Warsaw, Poland) was used to determine cell viability. Stained cells were analyzed using confocal laser scanning microscope (CLSM, Olympus Fluoview equipped with FV1000, Olympus Corporation, Tokyo, Japan).

#### 2.6.2. Cell Proliferation

**Figure 1:**
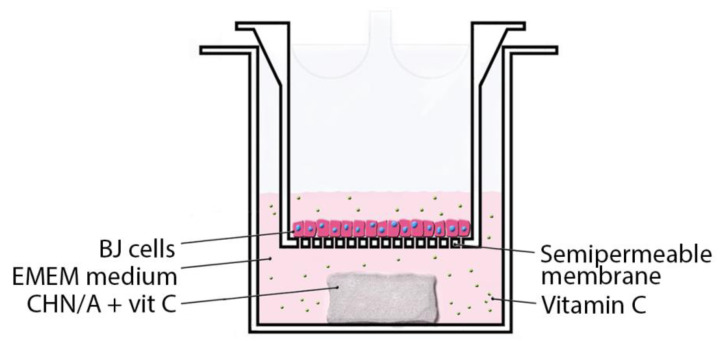
Schematic representation of the process of BJ culturing on the hanging cell culture inserts, providing two-compartment environment during cell culture tests.

To determine the effect of biomaterials on cell proliferation, hanging cell culture inserts (Millicell, Billerica, MA, USA) and BJ fibroblasts were used (Figure 1).

Application of cell culture inserts was necessary to get reliable results since biomaterials were designed so as to hinder cell attachment to their surfaces, enabling painless dressing removal. Prepared biomaterials samples (6 mm × 6 mm, 10 ± 1 mg) were stuck using agarose to the bottom of the well in a 24-multiwell plate. Then, cell culture inserts (12 mm diameter) were placed into the wells. In the next step, 1300 µL of EMEM medium was added outside the insert to the plate well, and 300 µL of BJ cell suspension at a concentration of 1 × 10^5^ cells per mL were added inside the insert. Control wells were prepared without biomaterials. Fibroblasts were cultured on the semipermeable insert membranes for 3 days and cell number was determined after 24 and 72 h using Cell Counting kit-8 (WST-8 test, Sigma-Aldrich Chemicals, Warsaw, Poland) according to the manufacturer protocol.

#### 2.6.3. Type I Collagen Production

To quantitatively assess collagen production by human fibroblasts, BJ cells were seeded identically to the proliferation assay and cultured for 5 days. One half of the medium was changed on the third day. ELISA kit specific to human type I collagen (Col I, EIAab ELISA kit, Wuhan, China) was used to determine the amount of produced collagen. Cell lysates were used for the ELISA test. They were obtained by application of ultrasonication and freeze-thaw cycles as was described in the following work [23]. BCA Protein Assay Kit (Thermo Fisher Scientific, Waltham, MA, USA) was used to normalize the amount of collagen per 1 mg of total cellular proteins. Additionally, the amount of collagen production was determined qualitatively using the immunofluorescence technique. BJ fibroblasts cultured on the insert membranes were fixed with 3.7% paraformaldehyde (Sigma-Aldrich Chemicals, Warsaw, Poland) and immunostained as described earlier [24]. Briefly, human specific anti-type I collagen (Col1a1/Col1a2) antibodies (Abnova, Taipei, Taiwan) were added to the fixed cells for 24 h at 6 °C. After the allotted time, cells on the membranes were washed with PBS and were incubated for 1 h with secondary antibodies conjugated to AlexaFluor647 (Abcam, Cambridge, UK). Finally, the cells were counterstained with DAPI (0.5 µg/mL) and observed using CLSM.

#### 2.6.4. Growth Factor and Matrix Metalloproteinase Production

BJ fibroblasts were seeded directly in the inserts as described in the proliferation assay. The cell line was cultured for 3 days, whereupon the amount of the matrix metalloproteinase-2 (MMP-2) and growth factors (GFs) was evaluated in cell culture supernatants. For this purpose, the ELISA kits specific to human platelet-derived growth factor-BB (PDGF-BB), transforming growth factor-beta 1 (TGF-β1), and MMP-2 (Biorbyt ELISA kit, Cambridge, UK) were used. Tests were performed according to the manufacturer protocol.

### 2.7. Statistical Analysis

All experiments were conducted in at least 3–4 independent tests or 3–4 independent biomaterial samples were tested. The results were presented as mean values ± SD. An unpaired *t*-test and one-way ANOVA, followed by Tukey′s test with statistically significant results considered at *p* < 0.05, were used to analyze statistical differences. Statistical analysis of the collected data was performed using GraphPad Prism 8.0.0 Software (GraphPad Software Inc., San Diego, CA, USA).

## 3. Results and Discussion

### 3.1. Preparation of the Biomaterial

The main function of the skin is to provide physical barrier from the external environment, protect the human body from pathogens, water loss, radiation, and temperature changes [25]. There are various types of commercial wound dressings available on the global market with their advantages and disadvantages, but there are only a few made from chitosan and agarose that were produced using freeze-drying method to obtain porous biomaterial for soft tissue regeneration [2,26,27]. In this study, an innovative method for the production of biomaterial made of agarose and chitosan with incorporated vitamin C was developed. The paper presents the production method and comprehensive characterization of new biomaterial for potential exuding wound management. Importantly, developed material was additionally enriched with different nontoxic concentrations of vitamin C in order to ensure the most appropriate conditions for cell viability and proliferation, thereby skin healing process. It is worth noting that in our previous research we have developed thin film/membrane with the same chemical composition (chitosan and agarose) [12,24]. The only difference in the production process was application of air-drying instead of freeze-drying. Surprisingly, this slight difference led to the fabrication of completely new biomaterial, having different properties. Air-dried CHN/A thin film was very supportive to the adhesion and proliferation of skin cells and had significantly lower exudate absorption capacity and elongation at break value compared to developed here foam-like highly porous biomaterial. Thus, developed previously CHN/A film was designed to be potentially used as an artificial skin substitute pre-seeded with the skin cells of the patient, whereas foam-like and superabsorbent biomaterial was developed to manage (as external dressing material) moderate to high exudate in the wounds.

**Figure 2:**
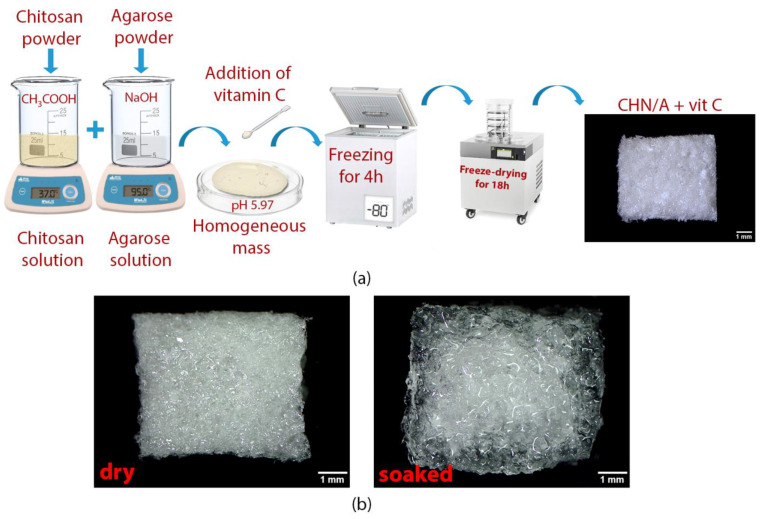
(**a**) A scheme presenting the production steps of porous vitamin C-enriched chitosan/agarose biomaterial; (**b**) Stereoscopic images presenting dry and hydrated dressing material.

Schematic representation of the biomaterial production is shown in Figure 2a. Developed production method allowed obtaining a novel highly porous foam-like biomaterial in the one simplified step. During production process, specific concentrations of agarose in NaOH and chitosan in CH_3_COOH were applied, ensuring mixture neutralization to pH approx. 6.0 without negative effect in the form of aggregation of chitosan, which precipitates at pH ≥ 6.5. Thus, the most important feature in the new production method were perfectly matched concentrations of the individual compounds, which prevented chitosan precipitation. The chitosan acetic acid solution was neutralized by alkaline agarose solution; as a result, the rinsing stage after production was skipped and the required nontoxic and slightly acidic pH was obtained.

After lyophilization, the biomaterial in an intact state resembled a foam-like dressing but, after liquid absorption, it changed physical state and formed a gel (Figure 2b), which is typical of hydrocolloid dressing. The pH of the produced biomaterial, measured at the stage of obtaining a homogeneous mass, was slightly acidic and equal to 5.97 for the biomaterial with the addition of vitamin C and to 5.99 for the biomaterial without ascorbic acid. A slightly acidic environment is the most appropriate for wound healing and skin tissue regeneration because of the energy that is required for human cell migration, proliferation, and extracellular matrix (ECM) synthesis [28]. Large numbers of contemporary in vitro and in vivo experiments revealed also that acidic pH is a key factor supporting antimicrobial barrier and reducing MMP activity in the chronic wounds since both the growth of a large number of pathogenic bacteria and MMP activity are inhibited at low pH [29].

### 3.2. Wettability and Porosity Characterization

**Figure 3:**
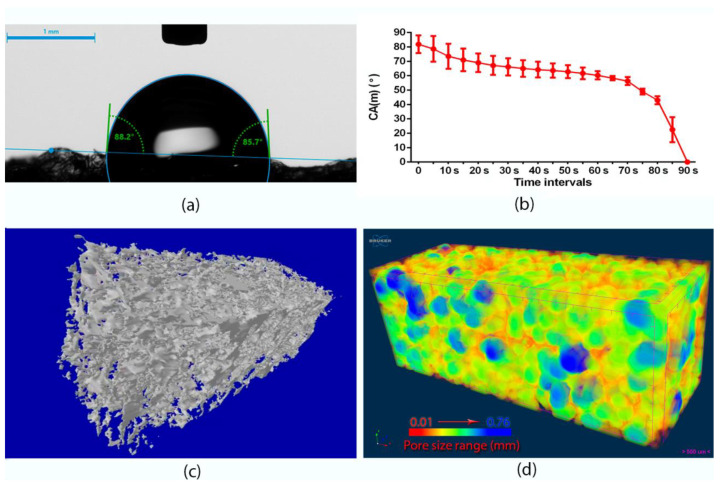
Wettability and porosity tests results: (**a**) Goniometer image presenting a contact angle of an ultrapure water droplet formed on the top surface of CHN/A biomaterial; (**b**) Graph showing static water contact angle measurements over time; (**c**) Three-dimensional model of porous biomaterial structure obtained with microCT; (**d**) MicroCT image presenting color-coded pore size distribution (from 0.01 to 0.76 mm).

To obtain biologically safe biomaterials that are in contact with the human body, some required surface properties must be achieved. Relevant interactions between tissue and the substrate depend on several parameters, including wettability, which highly influences biological response to biomaterial [30]. Wettability is an important parameter of the wound dressing since it affects eukaryotic cell behavior, bacteria adhesion, and protein adsorption [31]. Moreover, dressing biomaterial should possess hydrophilic properties in order to absorb high amounts of exudates. Therefore, static contact angle measurements were conducted to estimate interfacial tensions between two phases and evaluate material wetting characteristics. A conducted test revealed that the contact angle of an ultrapure water droplet formed on the top surface of the CHN/A biomaterial at time = 0 was equal to 88.13 ± 1.31 (Figure 3a), which proved the hydrophilic character of the biomaterial and its good wetting (that is considered when θ < 90°). Importantly, the water droplet on the CHN/A surface started spreading with time and was completely absorbed after approximately 1.5 min (θ ~ 0°), confirming high hydrophilicity of the material (Figure 3b).

The role of the wound dressing porosity is multiple because it ensures better nutrient transport, gas exchange, and improves absorption properties of the material [32]. The porosity of the developed chitosan/agarose biomaterial was estimated using microcomputed tomography. According to the conducted test, 82.4 ± 2.1% of total porosity was reached. Figure 3c shows three-dimensional model of CHN/A dressing obtained with microCT, presenting a highly porous structure of the produced biomaterial with color-coded pore size distribution, as shown in Figure 3d. Importantly, hydrophilic surface and highly porous structure are typical of the foam dressings, revealing relatively good exudate absorption ability. Thus, developed CHN/A biomaterial possesses very desirable features, allowing high amount of wound exudates to be absorbed and enabling good gas/oxygen exchange at the wound bed.

### 3.3. Mechanical Properties

**Figure 4:**
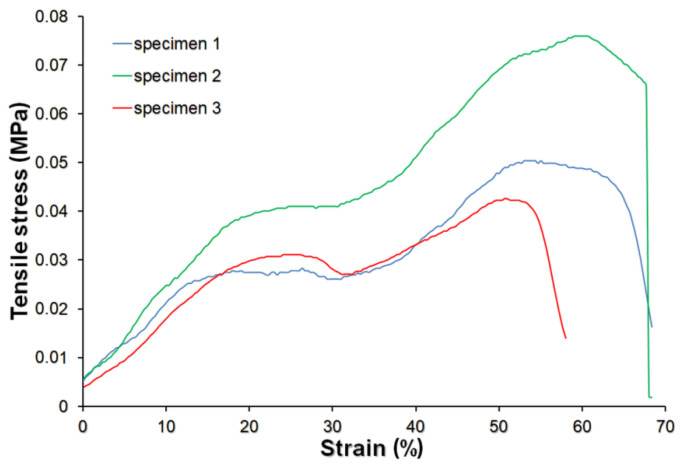
Tensile stress-strain curves obtained for three independent specimens of chitosan/agarose biomaterial.

The dynamic behavior of the biomaterials used as wound dressings plays an essential role in protective application use [33]. The chitosan/agarose biomaterial was prepared as an external highly absorbent wound bandage for repairing damaged skin tissue. Since biomaterial possessed a typical foam-like structure in its initial state and changed the structure (formed gel) after its soaking in a physiological fluids, mechanical properties were evaluated after CHN/A soaking in PBS solution for 1 h. A tensile test was performed and the obtained stress–strain curve is presented in Figure 4. The hydrated biomaterial showed the elastic-plastic behavior. The initial linear elasticity was caused by open cells wall bending and stretching in case of closed cells. In such a foam-type biomaterial, larger strains caused rotating of the cell edges towards the tensile axis, increasing the stiffness of the structure, which was demonstrated in the nonlinear stress increase above 10% of strain. Then, as the strain increased to about 20%, the cell walls rotated towards the tensile axis (by plastic bending), giving a yield point followed by a rising stress–strain curve, which was terminated by a fracture. Thus, the behavior of tested material corresponded well with the theory of cellular solids elaborated by Gibson and Ashby [34]. Estimated tensile strength value was relatively low and equal to 0.05 ± 0.02 MPa. The obtained value was lower than that that for the skin, whose tensile strength ranges from 1 to 32 MPa [35]. However, taking into account that calculated elongation at break was very high and equal to 75 ± 7.5%, it may be assumed that CHN/A material showed sufficient mechanical strength to provide the integrity and stability of the material during its use and stretching, especially given that external wound dressings are not subjected to high mechanical stress during the healing process. The biomaterial showed also low Young’s modulus value (0.15 ± 0.06 MPa), proving its high elastic deformations. Therefore, the elasticity and specific mechanical behavior of the obtained porous dressing would ensure its integrity and stability during the healing process even in the case of the high mobility of the patients.

### 3.4. Biodegradation Test

Ideally, external wound dressing should be biodegradable due to ecological issues and problems occurring with degradation of some wound care products. However, dressing material should be stable at the wound environment and not disintegrate during the healing process. Moreover, the potential degradation products cannot cause repression in cell proliferation and viability at the wound bed [36]. The biodegradation test was conducted in order to establish the biomaterial stability in enzymatic solutions (simulation of microenvironment occurring in the chronic and infected wounds) and non-enzymatic neutral solution (PBS, pH = 7.4). The degree of degradation was assessed on the basis of the changes in the pH and concentrations of reducing sugars in the degradation solutions collected every two weeks of the experiment.

Infection at the wound bed is an important problem because it retards healing, causing development of the chronic wound. Lysozyme production by the immune cells is one of the first basic ways of human immune system response to the bacteria infection. Thus, applied in this study, lysozyme-based degradation solution ideally reflected enzymatic microenvironment of infected wounds [37].

Matrix metalloproteinases (collagenases, membrane-type MMPs, stromelysins, etc.) are involved in abundant pathological and biological processes that are harmful or crucial for adequate wound healing [38]. In the presented study, the collagenase solution made of the mixture of MMP-1 and MMP-8 was used. Both mentioned collagenases are especially engaged in the skin wound regeneration process and their overexpression is observed in patients with chronic non-healing wounds [39]. Thus, application of the enzymatic solution containing collagenases in the degradation test simulated chronic wound microenvironment and allowed for the estimation of the stability of the porous biomaterial in an enzymatically active wound bed.

**Figure 5:**
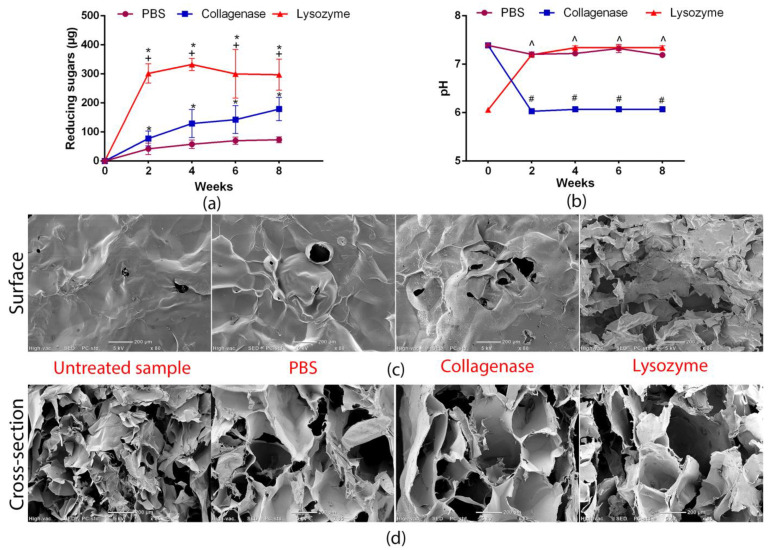
Results obtained with biodegradation test: (**a**) Concentrations of the reducing sugars in the degradation solutions (* statistically significant results compared to PBS, + statistically significant results compared to the solution with collagenases (mixture of MMP-1 and MMP-8); *p* value < 0.05, One-way ANOVA followed by Tukey′s test); (**b**) Changes in the pH values with time (# statistically significant results compared to the initial pH (7.4) of the collagenase solution, ^ statistically significant results compared to the initial pH (6.0) of the lysozyme solution; *p* value < 0.05, unpaired *t*-test); (**c**) SEM visualization of the biomaterial top surface after enzymatic and non-enzymatic degradation; (**d**) SEM visualization of the biomaterial cross-section after enzymatic and non-enzymatic degradation.

The performed test revealed that produced chitosan/agarose biomaterial was very stable in PBS (control, non-enzymatic solution) during 8-week incubation time since no significant changes in pH or the content of reducing sugars over time were noted (Figure 5a,b). Statistically significant results were obtained for lysozyme- and collagenase-treated samples. As can be seen in Figure 5b, the lysozyme solution increased pH from 6.00 to 7.35, whereas collagenase solution decreased pH from 7.39 to 6.06, indicating the degradation of the biomaterials. Lysozyme is known to cleave the β-1,4-glycosidic bonds in chitosan molecule [3]; however, it may also potentially cleave this bond in agarose. The pH rise during incubation in lysozyme solution may be caused by binding of H^+^ from slightly acidic buffer (pH = 6.0) and formation of –NH^3+^ groups in the chitosan molecule. Moreover, lysozyme-mediated degradation of the CHN/A biomaterial (cleavage of β-1,4-glycosidic bonds) could result in the greater amount of free –OH groups increasing the pH of the lysozyme solution [12,40]. In the case of collagenase solution, the decrease in the pH was most likely associated with the formation of acetic acid as a result of amide bond cleavage in the N-acetylglucosamine units present in the chitosan [12]. Consequently, the highest amount of reducing sugars was observed in the lysozyme samples, but statistically significant differences were also noted for the collagenase solution compared to the control solution (PBS) (Figure 5a).

Comparable observations were obtained with the SEM visualization since the lysozyme-treated sample noticeably had the most degraded surface (Figure 5c). In the case of collagenase-treated sample, single cracks were visible on the surface, indicating slight enzymatic biodegradation. The surface of the sample incubated in PBS solution remained almost unchanged compared to the untreated sample, which proved material high stability in non-enzymatic environment. The cross-section visualization (Figure 5d) of the biomaterial after incubation in degradation solutions revealed the presence of the largest pores in the lysozyme- and collagenase-treated samples. Slight changes (larger pores) in the cross-section structure were also observed for the PBS sample comparing to the untreated control.

Although reducing sugars release and changes in the pH indicated degradation of the biomaterial, it did not disintegrate after 8-week incubation on a shaker, proving its stability. Based on obtained results, it can be concluded that CHN/A biomaterial is very stable in neutral environment (PBS, pH = 7.4) and prone to enzymatic degradation with the greatest effect detected in the lysozyme solution. It should be noted that biodegradation test was performed for 8 weeks, whereas external dressings are usually changed every 2–5 days. Thus, it may be assumed that developed biomaterial should be stable in enzymatically active chronic and infected wound environment and maintain its integrity, allowing for easy dressing removal and change. Since CHN/A material is made of natural polymers and is prone to enzymatic degradation, it has features of easily biodegradable, eco-friendly wound care product.

### 3.5. Exudate Absorption Capacity

**Figure 6:**
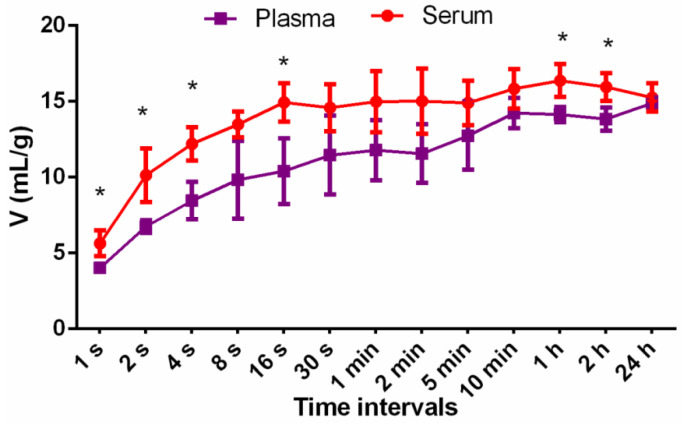
Exudate absorption capacity of the chitosan/agarose (CHN/A) biomaterial presented as a volume (mL) of serum/plasma absorbed by 1 g of the material (* statistically significant results compared to the plasma; *p* value < 0.05, unpaired *t*-test).

The process of exudate formation is a normal phenomenon during skin regeneration and its quantity depends on the type of the wound and varies starting from low to excessive exudation. It promotes epithelialization, cleans the wound, maintains the moisture at the wound bed, and contains growth factors and nutrients necessary for appropriate healing [41]. When a wound dries out, cells die complicating and lengthening the repair process. Therefore, maintenance of the moist wound environment and appropriate exudate management are the basic principles of the wound treatment and dressing selection [42]. In the case of wounds that produce excessive exudates, the uptake and retention of fluids along with adequate moisture balance are the most important step towards appropriate healing. Another issue concerns the necessity to minimize maceration of the skin area surrounding the wound [43]. The exudate absorption ability of the produced porous biomaterial was evaluated using human serum and plasma that mimicked wound exudates. The conducted test revealed that chitosan/agarose biomaterial had a high ability to uptake human fluids. The absorption equilibrium was reached after about 10 min (Figure 6). Due to a foam-like porous structure, 1g of the biomaterial absorbed about 15 mL of serum and 14 mL of plasma as can be seen in the graph in Figure 6. The differences in the absorption between the applied physiological fluids can be explained by the disparity in their density. It is most likely that fibrinogen content in the plasma hindered its diffusion into the biomaterial. High material absorption capacity was shown also as weight increase (*W_max_*) after 24 h incubation expressed in percentage. *W_max_* was estimated to be 1548 ± 26% after CHN/A immersion in plasma and 1542 ± 94% after immersion in serum. Thus, the initial dry biomaterial increased its weight 15 times after soaking in human fluids, indicating its high ability to uptake wound exudate. High absorption ability of the CHN/A was probably caused by its foam-like highly porous structure and hydrophilic character.

### 3.6. Vitamin C Release

**Figure 7:**
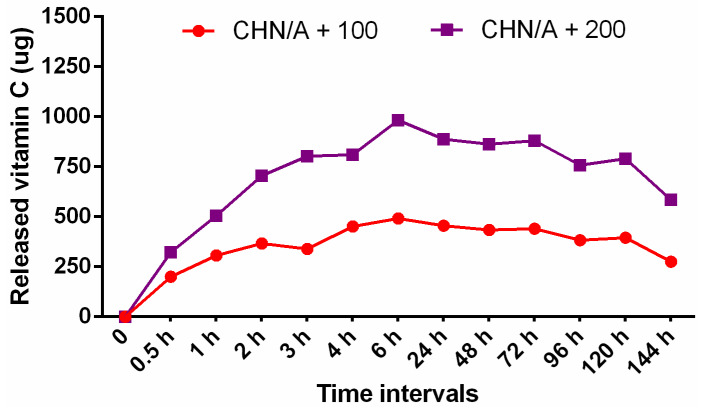
The profile of vitamin C release from CHN/A + 100 and CHN/A + 200 materials (CHN/A + 100 represents sample with the addition of 100 µg of vitamin C per 1 mL of biomaterial mass, CHN/A + 200 represents sample with the addition of 200 µg of vitamin C per 1 mL of biomaterial mass).

Human skin-dermis contains about 3–13 mg of ascorbic acid per 100 g of a wet weight. When it comes to human skin-epidermis, the amount is higher and stops between 6 and 64 mg per 100 g [15]. Numerous studies have shown that vitamin C stimulates wound healing mediators and simultaneously decreases the synthesis of pro-inflammatory mediators. It also supports fibroblasts migration and proliferation by altering the gene expression profiles of the cells [44]. Vitamin C deficiency results in the weakening of the collagenous structure causing impaired immunity and poor wound healing [45]. Considering this, it seems to be important to complete the ascorbic acid concentrations in the skin wound bed. In this context, biomaterials with incorporated vitamin C at 2 different concentrations (100 and 200 µg per 1 mL of biomaterial mass) were prepared to accelerate skin wound healing. A simple physical entrapment method was used to immobilize vitamin C in the biomaterial matrix. In the conducted test, vitamin C-enriched biomaterials (CHN/A + 100 and CHN/A + 200) showed gradual release of the ascorbic acid during the first 6 h of the experiment (Figure 7). Then, the plateau effect was observed and further detection of vitamin C concentration increase was not possible. A conducted test revealed that despite the different content of the vitamin C that was incorporated within the structure of the CHN/A dressing, the release profile for both samples was identical. Thus, ascorbic acid content did not affect the drug release profile from the biomaterial. Nevertheless, CHN/A + 200 sample that contained 2-fold more ascorbic acid, consequently released approx. 2-fold more vitamin C compared to CHN/A +100 at each time interval. Performed drug release test also showed that the whole content of vitamin C was released from both biomaterials during first 6 h regardless of the amount of ascorbic acid immobilized (100 or 200 µg per 1 mL of biomaterial mass). Thus, it can be concluded that the most effective process of skin wound healing would be achieved by changing the vitamin C-enriched CHN/A bandage every 1–2 days. It should be noted that typical foam dressings are changed every 2–3 days depending on the amount of exudate and type of the wound [46]. Importantly, developed production method of CHN/A dressing allows also for incorporation of other compounds or drugs that can potentially speed up or support the healing process.

### 3.7. Basic Biocompatibility Tests: Cytotoxicity and Cell Proliferation

**Figure 8:**
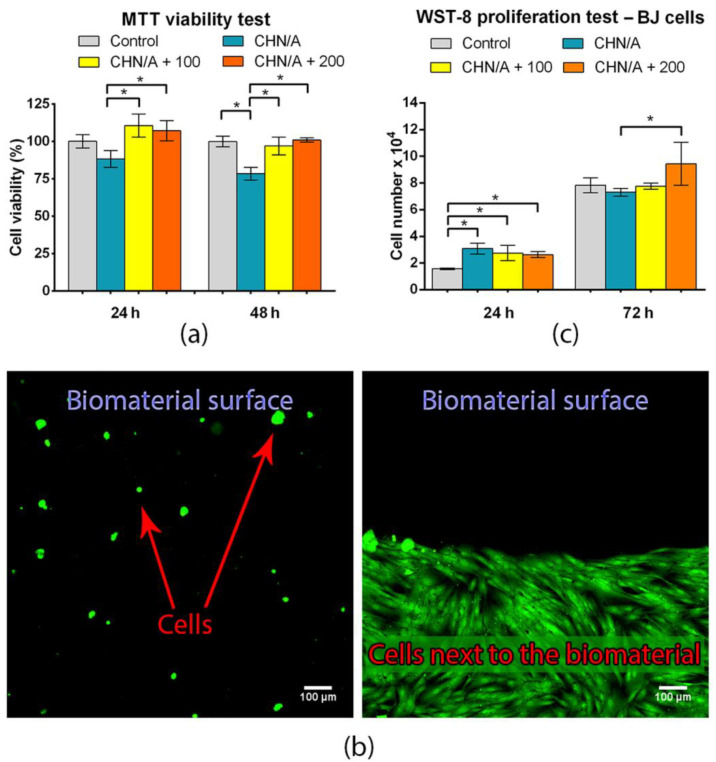
Basic biocompatibility tests for the chitosan/agarose biomaterial: (**a**) MTT cytotoxicity test conducted according to ISO 10993-5 [20] using extracts of the biomaterials and human skin fibroblasts (BJ cell line) (* statistically significant results between indicated groups, *p* value < 0.05, one-way ANOVA followed by Tukey’s test); (**b**) Live/Dead staining of the human skin fibroblasts cultured for 2 days on the CHN/A biomaterial and polystyrene surface next to the dressing (green fluorescent represents viable cells while red fluorescent represents dead fibroblasts); (**c**) Fibroblast proliferation determined in a two-compartment environment using cell culture inserts by colorimetric WST-8 assay (* statistically significant results between indicated groups, *p* value < 0.05, one-way ANOVA followed by Tukey’s test).

All biomaterials used as the wound dressings must be nontoxic to the human skin cells and provide an appropriate host response [47]. The cytotoxicity of the produced porous biomaterials was evaluated using MTT assay. The test was conducted according to the ISO 10993-5 [20] using 24 h extracts of the biomaterials. The cytotoxicity study was carried out using three different materials: without vitamin C (CNH/A) and with the addition of 100 (CHN/A + 100) and 200 (CHN/A + 200) µg of ascorbic acid per 1 mL of biomaterial mass. Cytotoxicity evaluation revealed that the produced chitosan/agarose bandages were nontoxic as the viability of BJ fibroblasts exceeded 78% for biomaterial without vitamin C and 97% for biomaterials with the addition of vitamin C at both time intervals of the experiment (Figure 8a). Although viability of cells exposed to CHN/A extract was significantly reduced compared to the control cells, viability still exceeded 70% indicating nontoxicity according to ISO 10993-5 [20]. Interestingly, extracts of the CHN/A with vitamin C addition significantly increased fibroblast viability, suggesting that ascorbic acid overcome negative effect of CHN/A material and supported cell viability.

Cytotoxicity of the CHN/A biomaterial was also estimated by culturing human skin fibroblasts cells (BJ) directly in contact with the sample. In the direct test, BJ cells were cultured for 2 days on the CHN/A biomaterial and visualized using Live/Dead staining. Obtained confocal microscopy images showed a few non-flattened, spherical, non-attached, but viable (stained green) skin fibroblasts on the surface of the CHN/A biomaterial (Figure 8b). In a conducted test, no dead cells (stained red) were observed. Moreover, typical growth of healthy fibroblasts with normal morphology was observed on polystyrene surface near the chitosan/agarose material. It clearly demonstrated that the biomaterial was nontoxic, but it hindered cell attachment and growth on its surface. In the case of temporary dressings that need to be changed every 2–3 days, it is very desired feature since it would ensure painless dressing removal due to the lack of supportive surface for cell adhesion. Thus, the produced biomaterial cannot function as a matrix (scaffold) supporting cell growth to generate artificial skin graft, but it can be used as an external highly absorbent and easily removable bandage to accelerate healing process.

Due to the fact that the obtained biomaterial did not support cell adhesion (Figure 8b), fibroblast proliferation was evaluated indirectly in a two-compartment environment using cell culture inserts (Figure 1). Vitamin C impact on the wound healing and cell proliferation is one of the most leading among all water-soluble vitamins [16]. Both the material without and with the addition of vitamin C slightly promoted the proliferation of human fibroblasts during first 24 h (Figure 8c). Statistically significant increase in cell number compared to the control well (without any biomaterial) was detected after 24 h for all kind of samples (Figure 8c). After 72 h, the greatest number of cells was observed on the insert membrane located in the well with the biomaterial with the higher concentration of vitamin C (CHN/A + 200), indicating a positive effect of the released ascorbic acid on cell proliferation (Figure 8c). In summary, performed basic biocompatibility tests showed that produced CHN/A biomaterial is nontoxic, does not support cell adhesion to its surface and slightly stimulates fibroblast proliferation during first 24 h, making it a good candidate to be used as an external wound dressing. Moreover, addition of vitamin C to the biomaterial (CHN/A + 200 sample) increases cell viability and significantly promotes cell proliferation compared to the CHN/A material.

### 3.8. Type I Collagen Production

**Figure 9:**
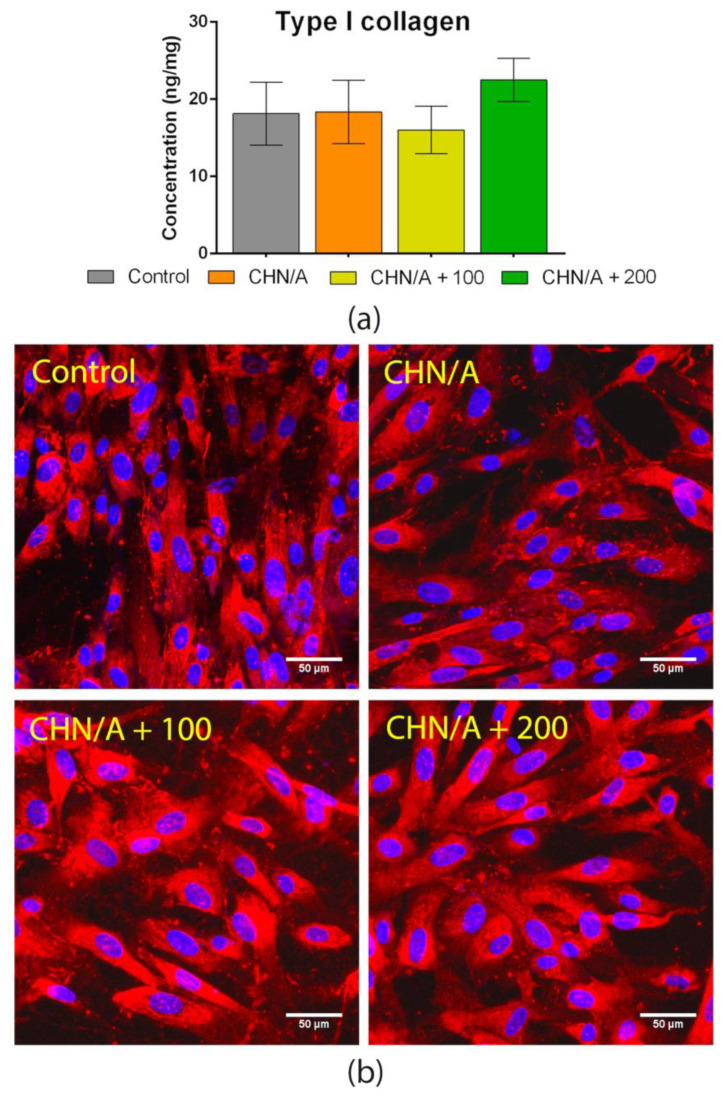
Type I collagen synthesis: (**a**) ELISA detection of the collagen in the cell lysates obtained after 5-day culture on the insert membranes in the presence of biomaterials; (**b**) Visualization of collagen deposited in the 5-day fibroblast culture that was performed on the insert membrane; immunofluorescent staining (type I collagen—red fluorescence, nuclei—blue fluorescence).

The fibrous structure of the ECM is basically made of stiff collagen and rubber-like elastin fibers. Dry weight of dermal matrix consists of 70–80% of type I collagen [48]. Therefore, proper collagen synthesis and its deposition by skin cells appear to be crucial in repairing the tissue damage. Abnormal scar formation as well as collagen defects are usually associated with vitamin C deficiency [16]. The stabilization of the collagen structure is provided by vitamin C as a cofactor for prolyl and lysyl hydroxylases [45]. The synthesis of type I collagen by human skin fibroblasts cultured in the presence of all tested samples (CHN/A, CHA/A + 100, CHN/A + 200) was assessed by an indirect test using cell culture inserts. As it can be seen in Figure 9a, the amount of produced type I collagen was comparable between all samples with the highest quantity in the case of CHN/A + 200 sample.

However, no statistically significant results were observed. Thus, it can be concluded that the addition of 200 µg ascorbic acid per 1 mL of biomaterial mass slightly increased collagen production, but the effect was insignificant. Vitamin C is engaged in collagen regulation and metabolism; thus, it seems to be important to deliver it to the wound bed [44]. Nevertheless, it should be noted that excessive collagen production may lead to unaesthetic scar formation and is an undesired phenomenon. Comparable deposition of collagen protein in ECM of fibroblast culture was also confirmed by CLSM observation (Figure 9b). Based on obtained results it may be concluded that developed CHN/A biomaterial does not hinder normal type I collagen deposition in ECM. Surprisingly, vitamin C-enriched variants of the dressing do not positively affect production of this protein.

### 3.9. GF and MMP-2 Production

**Figure 10:**
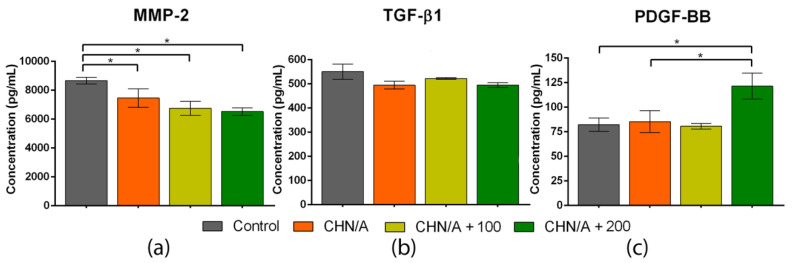
The effect of biomaterials on the production of matrix metalloproteinase-2 (MMP-2) and growth factors by human skin fibroblasts (BJ cell line) cultured for 3 days on the insert membrane assessed by ELISAs: (**a**) Release of the MMP-2 (* statistically significant results between indicated groups, *p* value < 0.05, one-way ANOVA followed by Tukey’s test); (**b**) Release of transforming growth factor-beta 1 (TGF-β1); (**c**) Release of platelet-derived growth factor-BB (PDGF-BB) (* statistically significant results between indicated groups, *p* value < 0.05, one-way ANOVA followed by Tukey’s test).

Wound re-epithelialization proceeds thanks to several closely related processes such as ECM deposition and its degradation due to the activity of matrix metalloproteinases (MMPs). High concentration and excessive activity of the MMPs are typical of chronic non-healing wounds [49]. MMP-2, which belongs to gelatinases group, is specially engaged in the wound healing by accelerating cell migration. Its overexpression was observed in chronic and normal diabetic wounds [39]. Chronic wounds are usually characterized by an unbalanced milieu. Importantly, the appropriate concentration of the various compounds (e.g., MMPs, GFs) at the wound bed is crucial as it enables the normal course of wound repair [50]. A great advantage of the dressing material is its potential usefulness in the treatment of many different wound types, including exudative, acute, and chronic wounds. Therefore, we conducted experiments aiming to evaluate the inhibitory or stimulatory effect of the developed biomaterials on the synthesis of various compounds that occur in the unbalanced milieu typical of chronic wounds. For this purpose, ELISAs for MMP-2, TGF-β1, and PDGF-BB were conducted. The study revealed that all variants of the CHN/A biomaterial significantly inhibited the synthesis of the MMP-2 (approx. 6500–7500 pg/mL) compared to the control (approx. 8700 pg/mL) (Figure 10a). Importantly, the higher vitamin C concentration was incorporated within the CHN/A structure, the stronger MMP-2 inhibition (with statistically significant differences) was noted.

Chronic wound environment is also characterized by imbalance in the concentration of various GFs. For instance, chronic pressure ulcers have reduced level of TGF-β and PDGF comparing to acute wounds. Similarly, lower PDGF expression is observed in chronic dermal ulcers compared to the acute ones [51]. It is suggested that the deficiencies of the mentioned compounds are accountable for chronicity of the wounds. The conducted study showed that human skin fibroblasts produced the comparable level of TGF-β1 in the presence of all tested samples and control insert (without biomaterial), as it can be seen in Figure 10b. In the case of PDGF-BB, its highest amount (approx. 120 pg/mL) was observed for the fibroblasts cultured in the presence of the CHN/A + 200 sample (Figure 10c). Cells cultured without biomaterial (control) and with CHN/A and CHN/A + 100 released similar amounts of PDGF-BB (approx. 80 pg/mL). It may be inferred that immobilization of the vitamin C within the CHN/A structure at the concentration of 200 µg per 1 mL of the biomaterial was sufficient to support PDGF-BB synthesis, which is essential for appropriate wound regeneration. Despite the lack of positive effect of the CHN/A + 200 on the TGF-β1 production, observed stimulation of the PDGF-BB secretion and inhibition of MMP-2 production clearly demonstrated its great potential to be used for the management of chronic non-healing wounds.

## 4. Conclusions

The novelty of the work focuses on a new production method that uses optimized and selected concentrations of individual components (polysaccharides) and their solvents to obtain highly porous structure of the biomaterial and slightly acidic pH (below 6), providing optimal skin regeneration and preventing the uncontrolled precipitation of the chitosan component, which is insoluble at pH ≥ 6.5. Within this study, it was demonstrated that a developed production method allows fabrication of highly absorbent and porous chitosan/agarose dressing biomaterial, possessing desired properties for accelerated healing and ensuring a suitable environment in the wound bed to support skin regeneration. Developed dressing possesses high porosity, hydrophilic structure, and great absorption ability, which enables the removal of excessive exudate from the wound and provides adequate moisture for the regeneration process. Although the material is prone to enzymatic degradation, it is stable in the chronic and infected wound microenvironment, maintaining its high integrity. Importantly, the surface of the developed chitosan/agarose biomaterial hinders fibroblast attachment, ensuring painless dressing removal. Moreover, vitamin C-enriched biomaterial has the ability to significantly promote fibroblast proliferation, decrease MMP-2 production and stimulate PDGF-BB synthesis, which is very desirable during chronic wound management. Thus, obtained results clearly showed that developed biomaterial possesses all necessary features of the external dressing for the management of exudate from both acute and chronic non-healing wounds. Importantly, the use of marine-derived biopolymers fits well into the green chemistry approach as they typically do not require aggressive solvents as synthetic polymers, are easily biodegradable, and their source is renewable. Thus, produced superabsorbent biomaterial has eco-friendly characteristics, having great potential to be translated into clinical applications. Nevertheless, in vivo tests on animal model must be performed to confirm its clinical usefulness.

## 5. Patents

The method for the production of the CHN/A dressing was claimed in the Polish Patent Office (patent application no. P.430457).

## Figures and Tables

**Table 1:** Biomaterials designations and their composition.

Biomaterial Designation	Composition
CHN/A	2% (*w*/*v*) agarose, 1.5% (*w*/*v*) chitosan
CHN/A + 100	2% (*w*/*v*) agarose, 1.5% (*w*/*v*) chitosan, vitamin C (100 ug per 1 mL of prepared homogeneous mass)
CHN + 200	2% (*w*/*v*) agarose, 1.5% (*w*/*v*) chitosan, vitamin C (200 ug per 1 mL of prepared homogeneous mass)

## Data Availability

The raw/processed data required to reproduce these findings can be obtained from the corresponding author (agata.przekora@umlub.pl) upon reasonable request.
